# Intravenous immunoglobulins improve live birth rate among women with underlying immune conditions and recurrent pregnancy loss: a systematic review and meta-analysis

**DOI:** 10.1186/s13223-022-00660-8

**Published:** 2022-03-11

**Authors:** Denise H. J. Habets, Kim Pelzner, Lotte Wieten, Marc E. A. Spaanderman, Eduardo Villamor, Salwan Al-Nasiry

**Affiliations:** 1 Department of Obstetrics and Gynecology, Maastricht University Medical Centre (MUMC+), Maastricht, The Netherlands; 2 Department of Transplantation Immunology, Maastricht University Medical Centre (MUMC+), Maastricht, The Netherlands; 3 GROW School for Oncology and Developmental Biology, Maastricht University, Maastricht, The Netherlands; 4 Department of Pediatrics, Maastricht University Medical Centre (MUMC+), Maastricht, The Netherlands

**Keywords:** RPL, IVIG

## Abstract

Intravenous immunoglobulin (IVIG) is increasingly used as a treatment for recurrent pregnancy loss (RPL) despite lack of clear evidence on efficacy. Recent data suggest IVIG might be more effective in a subgroup of women with an aberrant immunological profile. Therefore, a systematic review and meta-analysis of studies on the effectiveness of IVIG treatment on pregnancy outcome among women with RPL and underlying immunological conditions (e.g., elevated NK cell percentage, elevated Th1/Th2 ratio, diagnosis with autoimmune disorders) was conducted. Eight non-randomized controlled trials, including 478 women (intervention: 284; control: 194), met eligibility criteria. Meta-analysis showed that treatment with IVIG was associated with a two-fold increase in live birth rate (RR 1.98, 95% CI 1.44–2.73, P < 0.0001). The effect of IVIG was particularly marked in the subgroup of studies including patients based on presence of elevated (> 12%) NK-cell percentage (RR 2.32, 95% CI 1.77–3.02, P < 0.0001) and when starting intervention prior to or during cycle of conception (RR 4.47, 95% CI 1.53–13.05, P = 0.006). In conclusion, treatment with IVIG may improve live birth rate in women with RPL and underlying immune conditions. However, these results should be interpreted with caution as studies are limited by low number of participants and the non-randomized design, which represent seriously biases. Future randomized controlled trials in women with RPL and underlying immune conditions are needed before using IVIG in a clinical setting.

**Supplementary Information:**

The online version contains supplementary material available at 10.1186/s13223-022-00660-8.

## Introduction

Recurrent pregnancy loss (RPL), typically defined as two or more pregnancy losses, is both physically and psychologically burdensome for couples trying to conceive [1]. This condition is a frequent reproductive problem worldwide, affecting up to 1% of couples [2]. Despite extensive clinical and laboratory investigations of genetic, hormonal and anatomical factors, the majority of women with RPL have no discernible cause. Currently, there is a prevailing conviction that immunological aberrations may be at fault in women with RPL, as it is evident that the maternal immune system needs regulation to avoid rejection of the semi-allogenic fetus [3].

Among the different immunological aberrations potentially associated with RPL are changes in levels of regulatory T cells and Natural Killer (NK) cells, NK cell cytotoxicity, ratios of T helper cells and the presence of excessive autoimmune reactivity to self-antigens [4]. Auto-antibodies that have been associated with RPL include anti-thyroid, antiphospholipid, lupus anticoagulant, anticardiolipin, antinuclear, anti-ssDNA, anti-dsDNA, and anti-histone [5–7]. Furthermore, there is compelling evidence showing that women with RPL have significantly elevated Th1 (proinflammatory) to Th2 (anti-inflammatory) ratios and reduced levels of regulatory T cells compared to normal fertile controls [8, 9]. More recently, a study showed that women with RPL have significantly increased activated peripheral blood NK cell levels compared to normal fertile controls [10].

Intravenous immunoglobulin (IVIG) treatment has been broadly applied to suppress excessive immune activation in autoimmune diseases. IVIG has been shown to inhibit the pathological-activity of a large number of disease-associated autoantibodies [11], to downregulate NK cell killing capacity [12], and to inhibit Th1 cytokines [13]. The mechanisms of action of IVIG are complex and a single mechanism might not account for its therapeutic benefit. Although IVIG has been widely used as an immune-modulating agent for more than 30 years, little is known about the factors that predict the success of this therapy [11].

IVIG has frequently been used as a generic treatment strategy for all women with RPL despite lack of clear evidence of improving pregnancy outcomes [14, 15]. A recent metanalysis showed that women with unexplained reproductive failure who have abnormal levels of NK cells are more responsive to immunotherapy [16]. However, this study combined the effect of various modalities of immunotherapies on a combined group of spontaneously conceived and IVF pregnancies. Along these lines, IVIG might be more effective in a subgroup of women with spontaneously conceived RPL and underlying immune conditions.

This systematic review and meta-analysis aimed to investigate the effectiveness of IVIG on live birth rate in women with RPL and an underlying immune condition, and to identify the women who might benefit most from IVIG treatment through subgroup analysis.

## Methods

This study was performed in line with the Preferred Reporting Items for Systematic Reviews and Meta-Analysis (PRISMA) reporting guidelines. A standardized Patient, Intervention, Comparison, Outcome (PICO) question was formulated as follows: In women with immunological conditions and recurrent pregnancy loss (P), does the treatment with IVIG (I) increase the live birth rate (O) as compared to no treatment (C)?

### Sources and search strategy

A comprehensive literature search was undertaken using PubMed. The literature search was updated up to September 2020. Search terms were combined using ‘AND’ and included MeSH terms such as ‘Recurrent Pregnancy Loss’, ‘Intravenous Immunoglobulin’, ‘Cytotoxicity’, ‘Natural Killer Cells’, ‘T-Lymphocytes’, ‘Cytokines’, and ‘Live birth rate’ as well as free text words. The exact search strategy is shown in Additional file 1: Appendix S1. Narrative reviews, systematic reviews, case reports, letters, editorials, and commentaries were excluded, but read to identify other potential studies. Additional strategies to identify studies included manual review of reference lists from key articles that fulfilled the eligibility criteria, use of the “related articles” feature in PubMed, and use of the “cited by” tool in Web of Science and Google scholar.

### Eligibility criteria

Studies were selected based on the following criteria: (1) women were included when having three or more pregnancy losses and (2) women were included when studies determined an aberrant immunological profile (defined as elevated number of NK cells or T-lymphocytes, elevated Th1/Th2 ratio or presence of autoimmune diseases). Criteria had to be applicable for both the intervention and the control group. Furthermore, women in the intervention group had to be treated with IVIG. The primary effectiveness outcome was live birth rate. Studies without screening for immunological profile before initial IVIG administration were excluded. Furthermore, studies without a control group or with a control group defined as women with a healthy pregnancy (no immunological aberrations) or non-pregnant women were also excluded. When encountering multiple reports of the same underlying population, the publication with the largest population was included. Lastly, no restriction on age of women was applied, study selection was restricted to English-language and the search was not restricted to a specific publication date.

### Study selection and data collection process

The study selection process was performed by one investigator (KP) and critically reviewed by a second investigator (SE). After an initial screening of titles and abstracts, full texts of the potentially eligible studies were retrieved. Full texts were reviewed on their compliance with eligibility criteria and completeness of data by both investigators. Key data were extracted from eligible publications using a data extraction form.

### Assessment of risk of bias in included studies

We intended to use the Cochrane Risk of Bias (RoB) Assessment Tool for randomized controlled trials (RCTs) [17]. However, no RCTs were identified (see “Results”). The RoB In Non-randomized Studies of Interventions (ROBINS-I) tool was used to determine the risk of bias per included study [18]. RoB was scored by two independent investigators (KP and DH) on seven domains at three different time points. 1. Pre-intervention (bias due to confounding, bias in selection of participants of the study); 2. At intervention (bias in classification of interventions); 3. Post-intervention (bias due to deviations from intended interventions, bias due to missing data, bias in measurement outcomes, bias in selection of the reported results). Each domain was scored as either low risk, moderate risk, serious risk, critical risk or no information. An overall judgement of RoBs was also performed using the following criteria: (1) Low RoB: studies had low RoB in all domains; (2) Moderate RoB: studies had moderate RoB in at least one domain cut not serious or critical RoB in any domain; (3) Serious RoB: studies had serious RoB in at least one domain, but not critical RoB in any domain; (4) Critical RoB: studies had critical RoB in at least one domain; and (5) No information: when there was no clear indication that the study had serious or critical RoB and there was a lack of information in one or more key domain. Finally, the information on conflict of interest or any funding from commercial agencies was also recorded and evaluated.

### Statistical analysis

Included studies were combined and analyzed using comprehensive meta-analysis V3.0 software (Biostat Inc., Englewood, NJ, USA). The risk ratio (RR) with 95% confidence interval (CI) was calculated from the data provided in the studies. Due to anticipated heterogeneity, summary statistics were calculated with a random-effects model. This model accounts for variability between studies as well as within studies. To identify any study that may have exerted a disproportionate influence on the summary effect, we deleted studies one at a time in sensitivity analysis. Statistical heterogeneity was assessed by Cochran’s Q statistic and by the I^2^ statistic, which is derived from Q and describes the proportion of total variation that is due to heterogeneity beyond chance [19]. I^2^ was interpreted based on the following reference points: 25%, 50%, and 75%, representing low, moderate, and high heterogeneity, respectively [20]. We used the Egger’s regression test and funnel plots to assess publication bias. A probability value of less than 0.05 (0.10 for heterogeneity) was considered statistically significant.

Potential sources of heterogeneity were assessed through subgroup analysis and subgroups were compared by random effects (method of moments) meta-regression analysis [19]. The sources of heterogeneity analyzed were: (1) use of increased NK cell percentage (> 12%) as criterion for inclusion in the study; (2) Initiation of the intervention after pregnancy confirmation; (3) Initiation of the intervention prior to planned conception or during cycle of conception and (4) IVIG dosage/administration scheme.

## Results

### Study selection

The PRISMA flowchart of the included studies is shown in Additional file 1: Fig. S1. Out of 243 potentially relevant studies, 8 met eligibility criteria, all were non-randomized controlled trials.

### Characteristics of included studies

Characteristics of the included studies are summarized in Table 1. Studies were performed in Iran (2 studies), Spain (2 studies), USA (2 studies), Kuwait (1 study) and Italy (1 study). In total 478 participants were included in these studies: 284 women with an aberrant immunological profile and RPL in the intervention group (IVIG treatment) and 194 women with an aberrant immunological profile and RPL in the control group.

**Table 1:** Characteristics of the included studies

Study	Country	Non-randomized design	Definition RPL	Inclusion criteria	N	Intervention			Outcomes
						IVIG (mg/kg)	Cycles	Continued until (weeks of gestation)	
Ahmadi et al. (2019)	Iran	Prospective	≥ 3 PL before GA of 20 weeks	Age 18–40 years NK-cell number > 12%	78	400 (ns)	Every 4 weeks Initiated after pregnancy confirmation	30–32	Live birth
Jafarzadeh et al. (2019)	Iran	Prospective	≥ 3 PL before GA of 20 weeks	Age 18–41 years Immune system abnormality	94	400 (Sandoglobulin)	Every 4 weeks Initiation not further defined	32	Live birth
Mahmoud et al. (2004)	Kuwait/USA	Prospective	3 PL between GA of 6 and 22 weeks	Positive for APS	15	500 (ns)	Every 4 weeks Initiated after pregnancy confirmation	34	Live birth
Moraru et al. (2012)	Spain	Prospective	≥ 3 PL before GA of 20 weeks	NK-cell number > 12% NKT-like cells > 10%	24	400 (Privigen or Flebogamma)	Every 3–4 weeks Initiated after pregnancy confirmation	13	Live birth
						200 (Privigen or Flebogamma)	Every 4 weeks	35	
Perricone et al. (2008)	Italy	Prospective	≥ 3 PL	Systemic lupus erythematosus	24	500 (Flebogamma)	Every 3 weeks Initiated after pregnancy confirmation	33	Live birth
Ramos-Medina et al. (2014)	Spain	Retrospective	≥ 3 PL before GA of 20 weeks	NK-cell number > 12% NK-cell like number > 10%	121	400 (Privigen or Flebogamma)	Every 3 weeks Initiated after pregnancy confirmation	1st trimester	Live birth
						200 (Privigen or Flebogamma)	Every 4 weeks	35–36	
Stricker et al. (2005)	USA	Prospective	≥ 3 PL	Age > 28 years Abnormal immunologic tests including among others T-cell counts and NK-cell levels	64	200 (Venoglobulin-S)	Every 4 weeks Initiated 2 weeks prior to planned conception	26–30	Live birth
Winger et al. (2008)	USA	Retrospective	≥ 3 PL	Women with NK-cells > 15% or CD56 number > 12% were offered IVIG	58	400 (ns)	At least once during cycle of conception and/or at least once after pregnancy confirmation	–	Live birth

*GA* gestational age, *IVIG* intravenous immunoglobulin, *NK* natural killer, *PL* pregnancy loss, *RPL* recurrent pregnancy loss, *ns* not specified

Inclusion and exclusion criteria varied between the included studies. Ahmadi et al. [21], Moraru et al. [24], Ramos-Medina et al. [26] and Winger et al. [28] only included women with an NK cell number above or equal to 12% of total lymphocytes. Perricone et al. [25] only included patients with systemic lupus erythematous and antiphospholipid syndrome and Mahmoud et al. [23] only included patients who were positive for the antiphospholipid syndrome. Stricker et al. [27], Jafarzadeh et al. [22] and Ahmadi et al. [21] reported to exclude women with anatomic, infectious, genetic or endocrine aetiologies of RPL. Moraru et al. [24] reported to exclude women with infectious or lymphoproliferative diseases and Perricone et al. [25], Mahmoud et al. [23] and Winger et al. [28] did not report any exclusion criteria at all. Immunological abnormalities of included patients are further specified in Additional file 1: Table S1.

The included studies differed in IVIG dosage regimens and number of cycles. Ahmadi et al. [21] reported to initiate IVIG administration at time of a positive pregnancy test. IVIG administration was continued every 4 weeks during pregnancy until 30–32 weeks of gestation with a dosage of 400 mg/kg body weight. Mahmoud et al. [23] and Perricone et al. [25] followed a similar protocol. However, they used a higher dosage (500 mg/kg body weight) every 3–4 weeks until a gestational age of 33–34 weeks. Moraru et al [24]. and Ramos-Medina et al. [26] reported administration of 400 mg/kg body weight every 3 to 4 weeks until a gestational age of 13 weeks and afterwards patients were given a lower dose of 200 mg/kg body weight until 35 weeks of gestation. Stricker et al. [27] reported to initiate IVIG administration 2 weeks prior to planned conception with a dose of 200 mg/kg body weight and, after pregnancy was confirmed, IVIG administration was continued every 4 weeks until a gestational age of 26 to 30 weeks using the same dose. Winger et al. [28] reported that 400 mg/kg body weight was administered only once during the cycle of conception and/or at least once after a positive pregnancy test.

### Risk of bias within studies

Quality assessment of the included studies is shown in Additional file 1: Fig. S2. Overall, RoB was serious in 1 study and critical in 7 studies. RoB due to confounding and classification of intervention was serious in most studies. Most studies had concerns in RoB due to missing data. Furthermore, RoB during selection of participants (e.g. by screening for NK cell levels before initiation of IVIG treatment) was critical in most studies. Subgroup analysis based on the RoB was unfortunately not feasible due to the low total number of studies.

### Meta-analysis

**Fig. 1:**
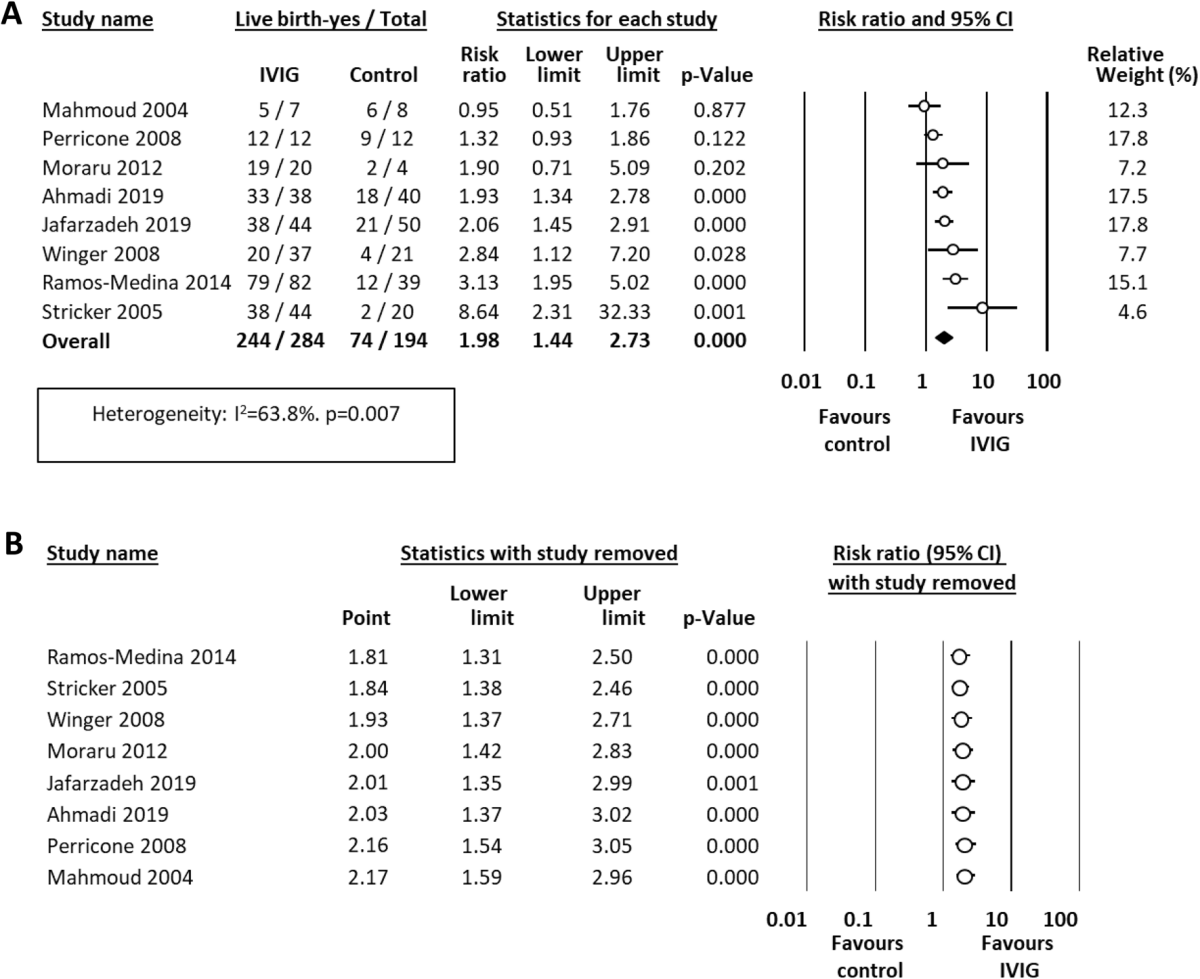
Meta-analysis on the effect of intravenous immunoglobulin treatment on live birth rate in women with immunological abnormalities and recurrent pregnancy loss or recurrent implantation failure. **A** Forest plot showing the risk ratio (RR) for live birth. A RR > 1 means that the event (live birth) is more likely to occur in the intervention than in the control group. **B** Sensitivity analysis showing the effect of removing one study at a time on the pooled RR. Each individual point represents the RR of the meta-analysis if the indicated study is excluded. For example, if the study of Ramos-Medina is excluded, the RR would become 1.81 (95% CI 1.31–2.50) in place of 1.98 (95% CI 1.44–2.73)

Results of the included studies are calculated as RR for live birth and summarized in Table 2. Meta-analysis showed that treatment with IVIG was associated with a significant increase in 5 out of 8 included studies and an overall significant improvement in live birth rate (8 studies, RR 1.98, 95% CI 1.44–2.73, P < 0.0001, Fig. 1A), although with a moderate heterogeneity (I^2^ = 58.9%). In sensitivity analyses, excluding one study at a time, the summary RR ranged from 1.81 (95% CI 1.31–2.50), when the study of Ramos-Medina et al. [26] was excluded, to 2.17 (95% CI 1.59–2.96), when the study of Mahmoud et al. [23] was excluded (Fig. 1B). Neither visual inspection of the funnel plot (Additional file 1: Fig. S3) nor the regression test of Egger (P = 0.362) revealed evidence of publication bias.

**Table 2:** Outcomes of the included studies

Study	N	Mean (SD) pregnancy duration (weeks)		Live birth events n (total n)			
		Intervention	Control	Intervention	Control	RR [95% CI]	p-value
Ahmadi et al. (2019)	78	39.1 (2.1)	38.3 (2.6)	33 (38)	18 (40)	1.93 [1.34, 2.78]	< 0.001
Jafarzadeh et al. (2019)	94	Not reported	Not reported	38 (44)	21 (50)	2.06 [1.45, 2.91]	< 0.001
Mahmoud et al. (2004)	15	Not reported	Not reported	5 (7)	6 (8)	0.95 [0.51, 1.76]	0.877
Moraru et al. (2012)	24	Not reported	Not reported	19 (20)	2 (4)	1.90 [0.71, 5.09]	0.202
Perricone et al. (2008)	24	37.5 (0.9)	37.2 (2.49)	12 (12)	9 (12)	1.32 [0.93, 1.86]	0.122
Ramos-Medina et al. (2014)	121	Not reported	Not reported	79 (82)	12 (39)	3.13 [1.95, 5.02]	< 0.001
Stricker et al. (2005)	64	Not reported	Not reported	38 (44)	2 (20)	8.64 [2.31, 32.33]	0.001
Winger et al. (2008)	58	37.2 (3.6)	38.8 (1.0)	20 (37)	4 (21)	2.84 [1.12, 7.20]	0.028

*NK* natural killer, *SD* standard deviation

We investigated the potential sources of heterogeneity through subgroup analysis. As shown in Table 3, when the 4 studies that used increased NK-cell percentage (≥ 12%) as criterion for inclusion were pooled [21, 24, 25, 28], meta-analysis showed a stronger association with live birth rate (RR 2.32, 95% CI 1.77–3.02, P < 0.0001) and heterogeneity disappeared (I^2^ = 0.0%). In contrast, meta-analysis of the 4 studies that did not use NK-cell percentage as criterion for inclusion [22, 23, 25, 27] showed a RR of 1.73 (95% CI 1.02–2.92) and high heterogeneity (I^2^ = 75.5%). However, meta-regression could not demonstrate a statistically significant difference in effect size between these two subgroups (P = 0.266).

**Table 3:** Subgroup analysis

Subgrouping criteria	No. of studies	Studies	Effect size			Heterogeneity	
			RR	95% CI	p-value	I^2^ (%)	p-value
Use of increased NK-cell percentage (> 12%) as criterion for inclusion	4	Ahmadi (2019), Moraru (2012), Ramos-Medina (2014), Winger (2008)	2.32	[1.77, 3.02]	< 0.0001	0.0	0.413
No use of increased NK-cell percentage (> 12%) as criterion for inclusion	4	Jafarzadeh (2019), Mahmoud (2004), Perricone (2008), Stricker (2005)	1.73	[1.02, 2.92]	0.041	75.5	0.007
Initiation of the intervention after pregnancy confirmation	5	Ahmadi (2019), Mahmoud (2004), Moraru (2012), Perricone (2008), Ramos-Medina (2014)	1.71	[1.16, 2.53]	0.007	67.7	0.015
Initiation of the intervention prior to planned conception or during cycle of conception	2	Stricker 2005, Winger 2008	4.47	[1.53, 13.05]	0.006	45.2	0.177

Time of initiation of the intervention was also used as subgrouping criterion (Table 3). In 5 studies [21, 23–26], treatment with IVIG was initiated after confirmation of pregnancy and the meta-analysis confirmed a moderate heterogeneity in this subgroup (I^2^ = 67.7%). In 2 studies [27, 28], IVIG was initiated prior to planned conception or during cycle of conception. Pooling of these two studies yielded a RR of 4.47 (95% CI 1.53–13.05) with a low heterogeneity (I^2^ = 45.2%). Meta-regression could not demonstrate a statistically significant difference (P = 0.071) between the effect size of the studies initiating treatment before or after pregnancy confirmation. Finally, although the dose of immunoglobulins had been chosen as a subgroup criterion, the great heterogeneity in this variable did not allow identifying clear patterns for subgrouping.

## Discussion

### Main findings

This systematic review and meta-analysis represent an updated overview of studies on the impact of IVIG treatment in women with RPL who were selected based on an aberrant immunological profile. Five of the eight included studies reported a significant increase in live birth rate (RR ranging between 1.93 and 8.64) among women who received IVIG treatment. The meta-analysis showed that the overall rate of live birth was twice as high in the women treated with IVIG than in the control group (RR 1.98). Although limited by the low number of studies, our data suggest that selection of patients based on the presence of a high percentage of NK cells or initiation of IVIG treatment before pregnancy may further improve pregnancy outcomes.

### Strengths and limitations

The main strength of our systematic review and meta-analysis was the use of strict selection and inclusion criteria. Only studies using IVIG intervention on women with RPL and underlying immune conditions and reporting an effect on live birth were included. This increased the homogeneity of the study population and the clinical applicability, as a trade-off for reducing the total number of included studies, which is an important limitation of our study.

A second limitation of the evidence provided by our meta-analysis is the non-randomized design of included studies, which is more susceptible to selection bias [29]. For example, some studies selected IVIG treatment by patient’s preference [28] or based on the number of previous miscarriages [21]. To date, the only available RCTs on the effect of IVIG were in a general group of women with RPL who were not pre-screened on immunological abnormalities and used variable IVIG administration protocols. As argued by Valentine and Thompson, the reason to include non-randomized studies in systematic reviews is the need to synthesize the best available evidence when no or few RCTs are available [29]. As IVIG treatment is already commonly being offered to RPL patients, mainly in private clinics, we believe this review and meta-analysis is of value to both patients and clinicians in refining the indication for treatment. Nevertheless, due to the significant bias in the included studies, the data presented here need to be interpreted with caution.

A third limitation was the moderate statistical heterogeneity across the included studies, which is further addressed using subgroup analyses. Interestingly, pooling the four studies using an elevated percentage of NK cells (> 12%) as inclusion criterion led to the disappearance of statistical heterogeneity. In addition, the subgroup analysis including the two studies in which IVIG treatment was initiated before pregnancy, also showed an increase in the effect size and a reduction in the statistical heterogeneity. Nevertheless, due to the low number of studies, the results of the subgroup analyses should be interpreted with caution and regarded as hypothesis generators for future research. Therefore, it would be necessary to perform RCTs in women with RPL and underlying immune conditions using a standardized protocol for IVIG treatment.

Lastly, substantial clinical heterogeneity was detected in the included studies. The criteria used for defining immunological alterations were variable: including women who were screened with a battery of immunologic tests before inclusion [27] and women with only a diagnosis of autoimmune diseases [23, 25]. However, sensitivity analysis by sequential omission revealed that results are robust against omission of these studies. If women with autoimmune diseases were excluded, IVIG treatment still resulted in a significant improvement in live birth rate. Moreover, the laboratory definitions of the immunological alterations were variable. Most studies use the cut-off value of ≥ 12% of lymphocytes for abnormally raised NK cell levels described by Kwak et al. [30], although peripheral NK cells are considered to have a physiological range between 5 and 29% of lymphocytes [31].

Future adequately powered studies should define normal and abnormal ranges and determine an aberrant profile, not only for NK cells but also for other immune factors that are clinically relevant for RPL, before they can be used as a diagnostic tool to study IVIG treatment.

### Interpretation

Successful pregnancy requires a well-balanced maternal immune system that maintains tolerant toward the foetus while it is still capable of building and adequate immune response against pathogenic microorganisms [32]. Since IVIG can modulate a wide variety of autoimmune and chronic inflammatory diseases and supress excessive and unwanted immune activation [33], it has been proposed to have an immune modulating effect in women with RPL, and especially in those with an aberrant immune profile. The meta-analysis by Christiansen et al. [15] failed to show a significant effect of IVIG on pregnancy outcome in the general group of women with recurrent miscarriage or recurrent implantation failure without pre-screening on immunological conditions. Our results showed that pre-screening women resulted in a two-fold increase in live birth rate, with a particularly beneficial effect in a subgroup of women pre-screened for NK cell percentages (RR = 1.99 and 2.32, respectively). These results are in line with a recent study of Woon et al., showing a potential benefit of IVIG in a carefully selected combined population of RIF and RM women with peripheral NK cell dysfunction [16].

Current guidelines, such as the American Society for Reproductive Medicine and the European Society of Human Reproduction and Embryology, do not recommend testing for immune abnormalities in women with RPL due to low level quality of evidence [34, 35]. However, our data suggests that pre-screening on immunological biomarkers such as NK cells might be valuable for selecting patients who might benefit from IVIG treatment despite the limited evidence provided here. NK cells are the most abundant leukocytes in early pregnancy decidua and, presumably, they have multiple functions in facilitating healthy pregnancy. First, maternal NK cells in the uterus can directly interact with foetal trophoblasts, allowing trophoblast cells to invade until a certain extent for proper implantation [36]. Furthermore, NK cells secrete an array of cytokines in the uterus that are important for angiogenesis and thus normal placental development. During pregnancy, spiral arterioles are transformed into high capacitance and low-resistance vessels. This vascular adaptation created by the foetal trophoblast is necessary to keep up with the nutritional demands of the growing fetus [37]. When implantation or vascular adjustments are insufficient due to altered uterine NK cell function, it could lead to the early loss of pregnancy [38]. Although to a lesser extent, T cells are also present in early pregnancy decidua and facilitate healthy pregnancy by a predominantly Th2‐type immunity [39]. Moreover, large numbers of regulatory T cells are generated during pregnancy and murine studies demonstrated that these are crucial for fetal survival [40, 41]. Given these multiple functions, it would be relevant to further explore the potential influence of IVIG on decidual NK and T cells.

In women with reproductive failure, it has become increasingly common to test peripheral blood lymphocytes based on the assumed similarities between lymphocytes in blood and the uterus [10, 42]. However, uterine lymphocytes are evidently less cytotoxic [37] and the recent identification of molecularly distinct subgroups of lymphocytes in human decidua [43] suggests that measuring peripheral blood lymphocytes may not provide relevant information on the characteristics of lymphocytes in the uterus. Understanding characteristics of uterine lymphocytes remains a major challenge, as it requires invasive sampling, correlation with menstrual cycle and histological standardization [44–46]. Therefore, the data on peripheral blood lymphocytes should be interpreted with caution as their function does not necessarily reflect that of their counterparts in the uterus.

## Conclusion

Although this systematic review and meta-analysis shows that IVIG improved live birth rate in women with RPL and underlying immune disorders, caution should be taken before offering IVIG as a treatment for reproductive failure. The included studies are potentially biased and limited by the low number and non-randomized design of trials. Our understanding of the immunogenic pathogenesis of RPL is still incomplete and further inquiry into the role of the immune system in RPL is needed to determine a specific biomarker to predict which women with reproductive failure will benefit from IVIG treatment. Even so, these data provide basis for future prospective RCT’s in women with RPL and underlying immune conditions using a standardized protocol for IVIG treatment initiated before pregnancy heretofore using IVIG in a clinical setting.

## Supplementary Information

**Additional file 1: Fig. S1**. PRISMA flowchart. **Fig. S2**. Assessment of bias using ROBINS-I tool for non-randomized studies. **Fig. 3S.** Funnel plot for publication bias.** Table S1.** Specification of immunological abnormalities of included patients .

## Data Availability

All data generated or analysed during this study are included in this published article.
