# Supplementary material for: Intravenous immunoglobulins improve live birth rate among women with underlying immune conditions and recurrent pregnancy loss: a systematic review and meta-analysis
Source: Allergy Asthma Clin Immunol. 2022 Mar 11;18:23. doi: 10.1186/s13223-022-00660-8 (PMC8917719; doi:10.1186/s13223-022-00660-8)
Supplement: Supplementary file 1 — Additional file 1: Fig. S1. PRISMA flowchart. Fig. S2. Assessment of bias using ROBINS-I tool for non-randomized studies. Fig. 3S. Funnel plot for publication bias. Table S1. Specification of immunological abnormalities of included patients . [file 13223_2022_660_MOESM1_ESM.docx]

# **Additional file 1**

# Appendix S1

**Full electronic search strategy for PubMed**

Searched from March 1971 to April 2019

(((((((((((("Abortion, Habitual"[Mesh]) OR Recurrent miscarriage) OR Recurrent pregnancy loss) AND "Killer Cells, Natural"[Mesh]) OR Natural killer cells) OR NK cells) OR NK cell cytotoxicity) OR TH1/TH2 ratio) OR T-lymphocytes) AND "Immunoglobulins, Intravenous"[Mesh]) OR Intravenous immunoglobulin) OR IVIG) AND "Pregnancy Outcome"[Mesh]

**240 Hits, 15.12.20**

Free terms used for search*.*

| **Population** | **Intervention** | **Outcome** | **Design** | **Setting** |
| --- | --- | --- | --- | --- |
| Women with  Recurrent miscarriage  Recurrent pregancy loss  Habitual abortion  AND  Cytotoxicity  Natural killer cells  T-lymphocytes  Cytokines  TNFa | Intravenous immunoglobulin  Intravenous IG  Intravenous antibodies  IVIG | Pregnancy outcome  Live birth rate  Miscarriage rate | RCT  Case-control study  Prospective study  Retrospective study  Cohort study | Clinical setting |

**Figure 1S (Supplementary Information)**

**
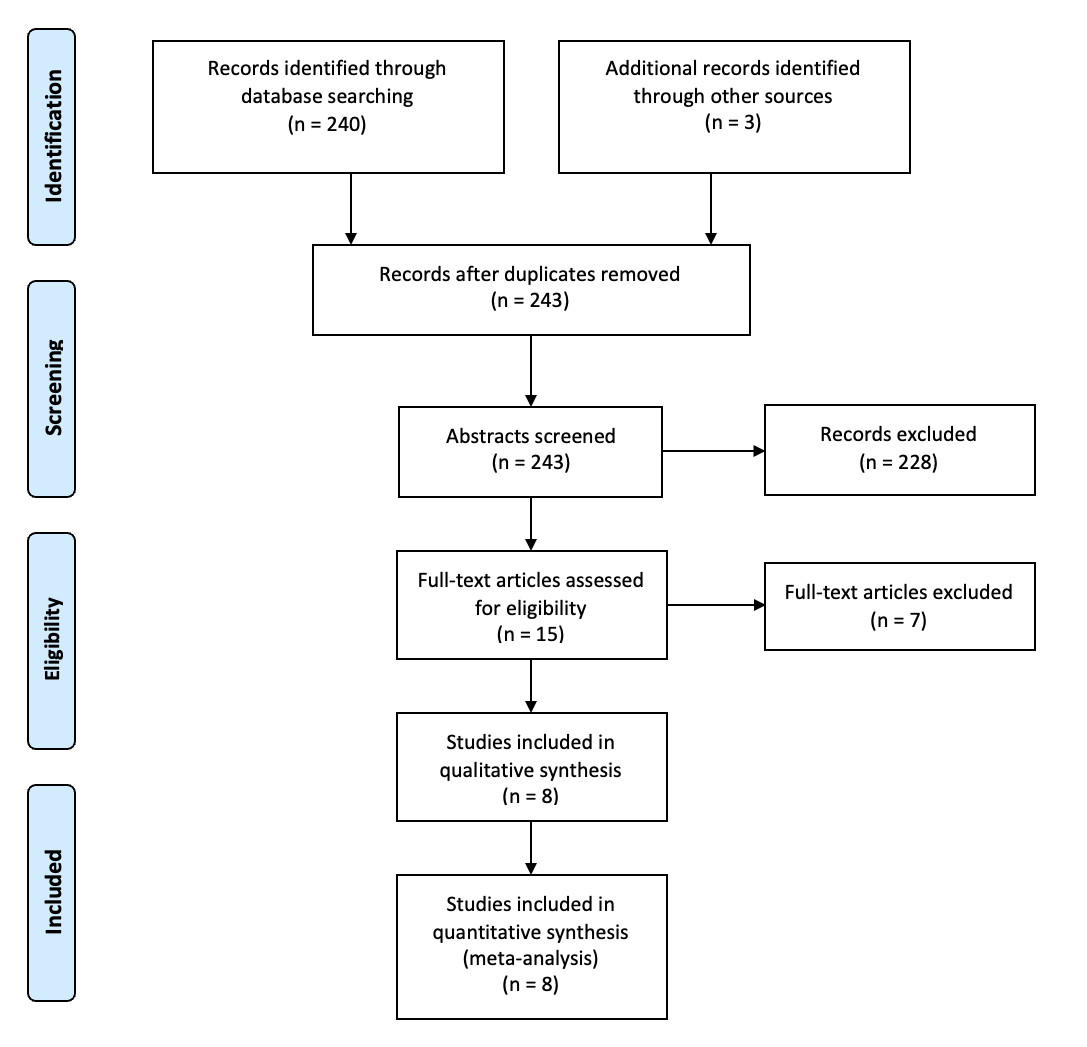
**

**Figure 2S (Supplementary Information)**

**Figure 3S (Supplementary Information)**

**
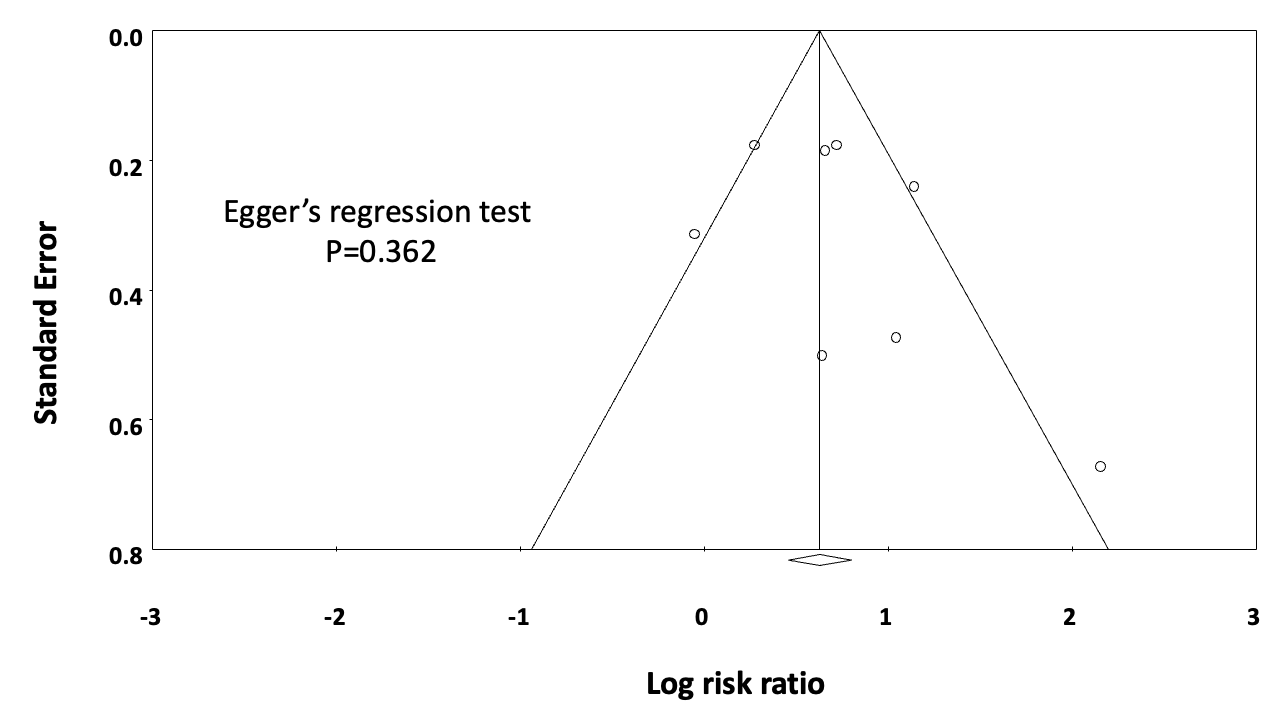
**

**Table S1 (Supplementary Information)**

*Table S1. Specification of immunological abnormalities of included patients*

| **Study** | **Inclusion criteria** | **Definition of abnormal lymphocyte markers** | **Screening for SLE** | **Screening for APS** |
| --- | --- | --- | --- | --- |
| Ahmadi et al.  2019 | Age 18-40 years  Elevated NK-cell number | NK-cell number > 12% | Not screened | Not screened |
| Jafarzadeh et al.  2019 | Age 18-41 years  Immune system abnormality | Not specified | Not screened | Not screened |
| Mahmoud et al.  2004 | Positive for APS | Not screened | Not screened | Positive for APS, not further specified |
| Moraru et al.  2012 | Elevated NK-cell number  Elevated NKT-like cells | NK-cell number >12%  NKT-like cells > 10% | Full fertility screening included determination of antinuclear antibodies; status not further specified for included women | Full fertility screening included determination of lupus anticoagulant and anti-beta-2-glycoprotein (IgG or IgM); status not further specified for included women |
| Perricone et al.  2008 | Systemic lupus erythematosus | Not screened | Screening included determination of antinuclear antibodies; disease activity was scored using lupus activity index-pregnancy scale (LAI-P; range 0-2.6); status not further specified for included women | Not screened |
| Ramos-Medina et al. 2014 | Elevated NK-cell number  Elevated NK-cell like number | NK-cell number > 12%  NK-cell like number >10% | Screening included determination of antinuclear antibodies; status not further specified for included women | Screening included determination of anticardiolipin, anti-beta-2-glycoprotein I (IgG and IgM) and lupus anticoagulant; status not further specified for included women |
| Stricker et al.  2005 | Age > 28 years  Abnormal immunologic tests including among others T-cell counts, NK-cell levels and different antibodies | NK-cell levels, CD4 and CD8 T-cell counts | Screening included determination of antinuclear antibodies; status not further specified for included women | Screening included determination of antiphospholipid antibody (IgG, IgA, IgM); status not further specified for included women |
| Winger et al.  2008 | Elevated NK-cell number  Elevated CD56-cell number | NK-cells > 15%  CD56-cell number > 12% | Not specified | Not specified |
